# Anti-Inflammatory Effects of Alphitolic Acid Isolated from *Agrimonia coreana* Nakai Extracts Are Mediated via the Inhibition of I_CRAC_ Activity in T Cells

**DOI:** 10.3390/ijms242417309

**Published:** 2023-12-09

**Authors:** Su Jin Park, Jin Seok Lee, Yu Ran Nam, Ji Min Lee, Dae-Won Ki, Bong-Sik Yun, Seong Woo Choi, Nhung Thi Hong Van, Joo Hyun Nam, Hyun Jong Kim, Woo Kyung Kim

**Affiliations:** 1 Department of Physiology, Dongguk University College of Medicine, 123 Dongdae-ro, Gyeongju 38066, Republic of Korea; sujin900710@gmail.com (S.J.P.); e_jimin@naver.com (J.M.L.); physiolcsw@dongguk.ac.kr (S.W.C.); vthn0295@gmail.com (N.T.H.V.); ferinus@gmail.com (J.H.N.); 2 Channelopathy Research Center (CRC), Dongguk University College of Medicine, 32 Dongguk-ro, Ilsan Dong-gu, Gyeonggi-do, Goyang 10326, Republic of Korea; youngshine80@naver.com; 3 Department of Internal Medicine, Graduate School of Medicine, Dongguk University, 27 Dongguk-ro, Ilsan Dong-gu, Gyeonggi-do, Goyang 10326, Republic of Korea; ggargo@daum.net; 4 CIPA KOREA Inc. 755-27, Gobong-ro, Gyeonggi-do, Paju-si 10911, Republic of Korea; 5 Division of Biotechnology and Advanced Institute of Environment and Bioscience, College of Environmental and Bioresource Sciences, Jeonbuk National University, Gobong-ro 79, Iksan 54596, Republic of Korea; dwki0916@gmail.com (D.-W.K.); bsyun@jbnu.ac.kr (B.-S.Y.)

**Keywords:** ORAI, *Agrimonia coreana*, *Agrimonia pilosa*, CD4^+^ T cell, alphitolic acid, anti-inflammation, CRACs (Ca^2+^ release activated Ca^2+^ channels), stromal interaction molecule 1 (STIM1)

## Abstract

*Agrimonia pilosa* Ledeb., an important medicinal herb in traditional East Asian medicine, is primarily used to treat abdominal pain, dysentery, and hemostasis. There are ten other reported species of *Agrimonia* plants, including *Agrimonia coreana* Nakai—a naturally growing species in South Korea—and *Agrimonia eupatoria* Linn. Although recent studies have isolated numerous active constituents and investigated their effects, the medicinal utility of this herb is not yet fully explored. Through patch-clamp recording, a previous study reported that *Agrimonia* plant extracts inhibit the function of Ca^2+^ release-activated Ca^2+^ channels (CRACs). Herein, we aimed to identify and isolate the main compounds in *A. coreana* responsible for CRAC inhibition while assessing the anti-inflammatory effects mediated by this inhibition. We demonstrated for the first time that alphitolic acid isolated from *A. coreana* has a dose-dependent inhibitory effect on CRAC activity and, thus, an inhibitory effect on intracellular calcium increase. Furthermore, analysis of human CD4^+^ T cell proliferation via the carboxyfluorescein diacetate succinimidyl ester method revealed that alphitolic acid inhibited T cell proliferation in a concentration-dependent manner. Our findings provide a theoretical basis for the potential therapeutic use of alphitolic acid in the treatment of inflammatory diseases.

## 1. Introduction

Inflammation is a defense mechanism against tissue damage caused by numerous factors in response to stimulants arising from the internal and external environments, including invading pathogens, such as viruses, bacteria, and fungi. Most direct causal effectors of inflammation are produced by immune cells. Further, although immune cells, such as T cells, protect the host by neutralizing diverse external pathogens, their excessive activity can cause disease development. For example, autoimmune diseases, such as rheumatism, are caused by the excessive activity of helper T type 1 cells, whereas the excessive activity of helper T type 2 cells leads to allergic diseases [1,2]. The T cell-mediated immune responses are initiated by increased intracellular calcium concentrations via calcium entry through ion channels, resulting in T cell proliferation and differentiation and cytokine secretion. Ca^2+^ release activated Ca^2+^ channels (CRACs) have a crucial role in calcium influx into T cells [3]. In particular, store-operated calcium entry (SOCE) is a specific type of calcium influx through CRACs. SOCE is initiated following T cell receptor (TCR) and co-receptor stimulation, leading to phospholipase C-γ1 (PLCγ1) activation, which triggers the hydrolysis of phosphatidylinositol-4,5-bisphosphate (PIP2) into inositol-1,4,5-triphosphate (IP3) and diacylglycerol. Subsequently, IP3 binds to the IP3 receptor in the endoplasmic reticulum (ER)—an intracellular calcium storage compartment—causing a decrease in ER calcium levels. This depletion triggers the binding between stromal interaction molecule 1 (STIM1) in the ER and ORAI1 ion channels in the cell membrane, effectively opening the ion channels and enabling the influx of calcium into the cell. SOCE is pivotal in regulating intracellular calcium signaling and activation of downstream pathways, including the Ca^2+^-dependent kinase-calmodulin (CaMK) and calcineurin (CaN) pathways and the nuclear translocation of the transcriptional regulator nuclear factor of activated T cell (NFAT). These events ultimately lead to the upregulation of target genes critical for innate and adaptive immunity [3,4].

CRACs also play a key role in most immune cells and, thus, participate in various immune mechanisms from phagocytosis and B cell activation to degranulation [3,5,6]. Abnormal functioning of CRACs has been linked to severe combined immunodeficiency and various other medical conditions, including allergies, atherosclerosis, and inflammatory bowel disease. Additionally, CRACs reportedly influence cancer cell proliferation and metastasis [7,8,9,10,11]. Accordingly, increased attention has been focused on substances that can modulate the activity of these channels [12]. In particular, curcumin, vitexin, alpha-mangostin, and fargesin exhibit CRAC inhibitory and anti-inflammatory effects [13,14,15,16,17]. The ongoing research related to these natural substances holds promise for developing novel compounds capable of inhibiting CRACs, contributing to the treatment and prevention of myriad diseases [12,18].

*Agrimonia coreana* Nakai is a perennial plant of the Rosaceae family and a species of the same genus as *Agrimonia pilosa*. Although these species have slight morphological variations, their active compounds are relatively conserved [19]. Nevertheless, few studies have examined the anti-inflammatory effects or the constituent compounds of *A. coreana*, a traditional medicinal herb used in East Asia, including Korea, Japan, China, and India, and listed in the Korean Herbal Pharmacopoeia under the name Yong-A-Cho. Records show that *A. coreana* has mainly been used for detoxification, alleviating fever, and treating enteritis, malaria, and hemostatic disorders. Moreover, it is used in traditional Chinese medicine for hemostatic, anti-malarial, detoxifying, and anti-tumor effects [20,21]. However, other medicinal effects of *A. coreana* are being investigated, with reports on anti-oxidant, anti-inflammatory, and anti-viral properties [22,23,24,25]. Furthermore, *A. coreana* contains myriad active compounds from flavonoids to triterpenes and their glycosides, isoflavones, isocoumarins, phloroglucinol derivatives, tannins, and organic acids, comprising 252 constituents, with numerous associated ongoing studies [20].

Alphitolic acid (ALA) is a pentacyclic triterpene constituent commonly isolated from *Alphitonia petriei* (Rhamnaceae), a tropical tree that naturally grows in areas from the north of New South Wales on the east of Australia to the Torres Straight in Queensland. However, ALA has been isolated from various other plants, including *Ziziphus jujuba* [26,27]. The numerous reported therapeutic effects of ALA range from anti-inflammatory and antioxidant to NO inhibition and anti-cancer activities [28,29,30]. Meanwhile, few studies have evaluated the anti-inflammatory effect within the context of ion channels.

Herein, we evaluated the anti-inflammatory effects of *A. coreana* via CRAC inhibition. To this end, we identified the main active constituent in *A. coreana* responsible for inhibiting CRAC activity and verified whether it elicited anti-inflammatory effects. The results of this study provide evidence for the potential application of *A. coreana*-derived ALA in the treatment of inflammatory diseases.

## 2. Results

### 2.1. Preparation of A. coreana Extracts and Isolation of Active Fractions

**Figure 1:**
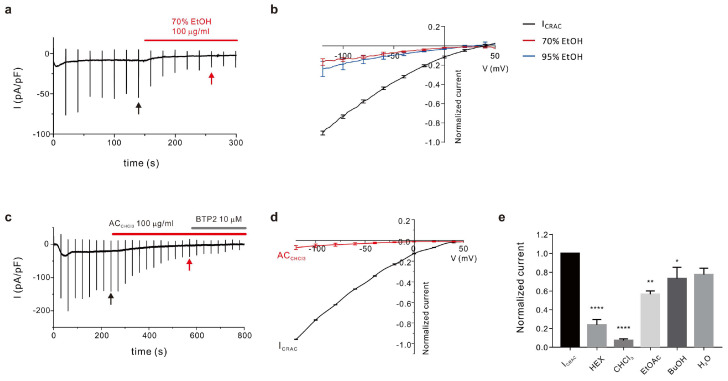
Column fractions for evaluating the I_CRAC_ inhibition effects, and isolating the active constituents, of *A. coreana.* The inhibitory effects of *A. coreana* extracts prepared using 70% and 95% ethanol (AC_70ext_ and AC_95ext_, respectively) on I_CRAC_. (**a**) Representative chart trace of the I_CRAC_ activity inhibition caused by AC_70ext_; the concentration at treatment was 100 mg/mL. (**b**) Current–voltage (IV) relationship curve of the inhibitory effects of AC_70ext_ and AC_95ext_; I_CRAC_ inhibition was detected at 100 mg/mL in each case. (**c**) Representative chart trace of the I_CRAC_ inhibition caused by the CHCl_3_ fraction of *A. coreana* extract (AC_CHCl3_); the concentration at treatment was 100 μg/mL. BTP2 was used as the control for comparing the inhibition rates, and its concentration at treatment was 10.0 μM. (**d**) IV curve of the inhibitory effects of AC_CHCl3_; the concentration at treatment was 100 μg/mL. (**e**) I_CRAC_ inhibitory effects of various *A. coreana* fractions. * *p* < 0.05, ** *p* < 0.01, **** *p* < 0.0001.

We prepared *A. coreana* extracts using 70% ethanol (EtOH) and 95% EtOH extracts (AC_70ext_ and AC_95ext_, respectively) from the collected *A. coreana* samples to determine the active constituent of *A. coreana* with inhibitory effects on CRAC activity. The yields of the two extracts were 10.85% and 4.25%, respectively, with AC_70ext_ showing a higher yield than AC_95ext_. Importantly, 95% ethanol and 70% ethanol have different polarities; hence, the carbohydrates, the flavonoid glucuronide (or glycoside), and tannin contained in *A. coreana* were relatively well soluble in 70% ethanol, whereas the lipids and terpenes were primarily extracted with 95% ethanol. As *A. coreana* is rich in flavonoids, the yield was expected to be higher with a 70% ethanol solvent. Analysis of the inhibition of CRAC (I_CRAC_) current using the two extracts revealed a stronger inhibitory effect with AC_70ext_ (81.27 ± 3.880%) than with AC_95ext_ (73.23 ± 8.790%; Figure 1a,b). This extract was separated into *n*-hexane (HEX), chloroform (CHCl_3_), ethyl acetate (EtOAc), butanol (BuOH), and water fractions.

Each fraction was evaluated for its inhibitory effect on I_CRAC_; a stronger effect was observed with HEX (76.02 ± 5.654%) and CHCl_3_ fractions (92.84 ± 1.901%) than with EtOAc fractions (43.31 ± 3.439%; Figure 1c–e). It was thus postulated that, in *A. coreana*, the main active constituent responsible for the anti-inflammatory effect via I_CRAC_ inhibition was a nonpolar compound in the HEX and CHCl_3_ fractions.

**Figure 2:**
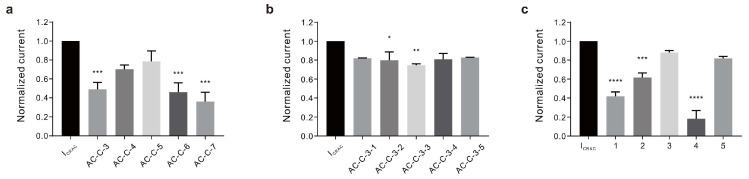
Fractionation of AC_70ext_ and I_CRAC_ inhibition. The inhibitory effects of *A. coreana* crude and column-purified fractions on I_CRAC_ are shown. (**a**) I_CRAC_ inhibition elicited by five of the seven fractions isolated using the silica gel column. (**b**) I_CRAC_ inhibitory effects of the five fractions, obtained after further separation using Sephadex LH-20 column. The solvent in the mobile phase was a 1:1 mixture of CHCl_3_ and MeOH. (**c**) I_CRAC_ inhibitory effects of the next five purified fractions obtained from ODS MPLC. Abbreviations: BTP2, 3,5-bis(trifluoromethyl) pyrazole derivative; HEX, hexane; CHCl_3_, chloroform; EtOAc, ethyl acetate; BuOH, butanol; H_2_O, water; MeOH, methanol; MPLC, medium-pressure liquid chromatography. * *p* < 0.05, ** *p* < 0.01, *** *p* < 0.001, **** *p* < 0.0001.

**Figure 3:**
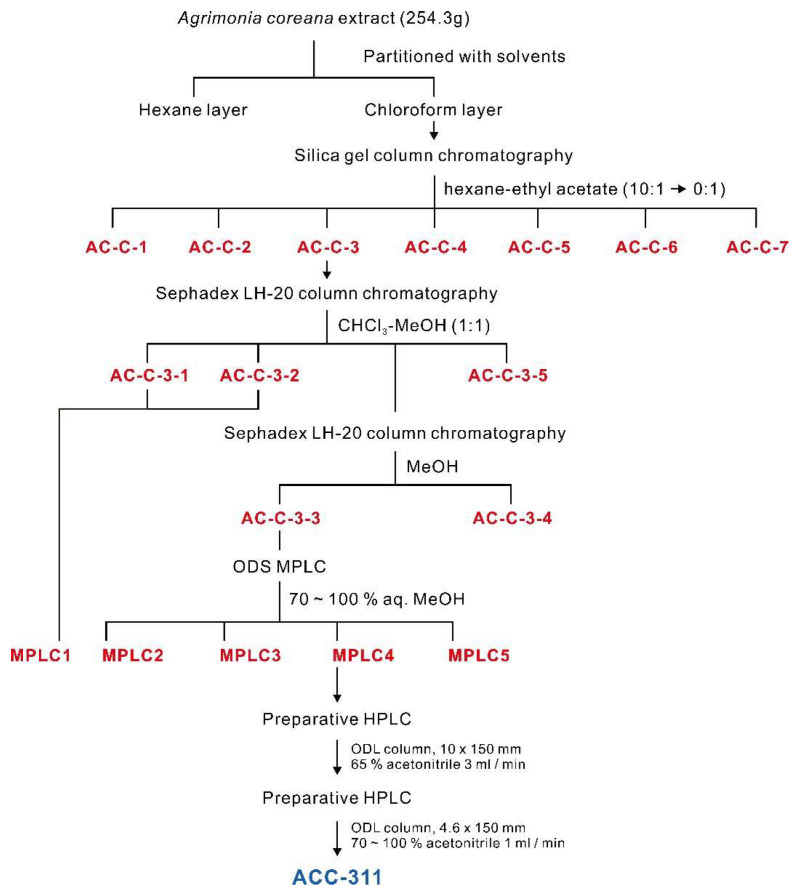
Schematic diagram of the isolation of purified compounds using different columns from AC_70ext_.

Therefore, to isolate and purify the active constituent in the CHCl_3_ fraction with the highest activity, silica column chromatography was performed on the CHCl_3_ layer to obtain seven fractions (AC-C-1 to AC-C-7). AC-C-3 (50.96 ± 7.190%), AC-C-6 (53.98 ± 9.776%), and AC-C-7 (63.91 ± 9.654%) exhibited inhibitory effects on I_CRAC_ (Figure 2a). We then assessed the fraction with the highest activity (AC-C-7) to identify the active ingredients. However, sufficient yields were not obtained to isolate the ingredients. Therefore, we selected AC-C-3, which had the second strongest activity, and confirmed the effect. Through Sephadex LH-20 column chromatography, the AC-C-3 fraction was further separated into five fractions (AC-C-3-1 to AC-C-3-5). The observed inhibitory effects of these five fractions on the I_CRAC_ were negligible compared with those of the previously obtained fractions. Nevertheless, the fraction with the most robust effect was AC-C-3-3 (25.40 ± 1.414%), which was used to perform preparative ODS medium-pressure liquid chromatography (MPLC) and for further fractionation into five groups (Figure 2b). Among these groups, No. 4 elicited the strongest effect (81.86 ± 8.631%; Figure 2c). The details of this fractionation are presented in the schematic diagram of Figure 3, and the I_CRAC_ inhibition results for each fraction are presented in Table 1.

### 2.2. Structural Analysis of ALA Using Spectroscopic Methods

**Figure 4:**
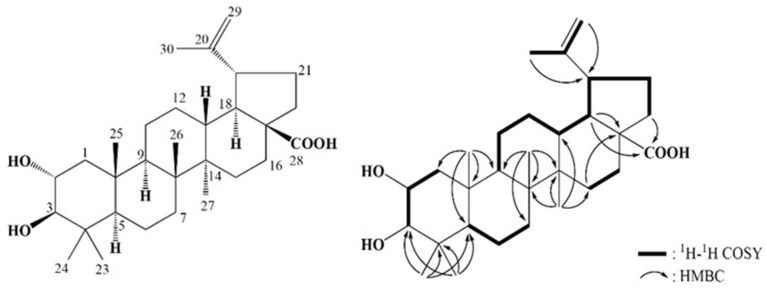
Chemical structure and two-dimensional NMR correlations of alphitolic acid (**1**).

We performed a high-performance liquid chromatography (HPLC) analysis and detected two peaks in Group 4 (ACC-311; 2.8 mg). We first isolated the active constituents of *A. coreana* related to its anti-inflammatory activity. The chloroform-soluble portion, exhibiting anti-inflammatory activity, was subjected to open column chromatography, i.e., MPLC and HPLC, to obtain ACC-311. The molecular weight of ACC-311 was established as 472 by quasi-molecular ion peaks at *m*/*z* ratios of 470.9 [M−H]^−^ and 942.9 [2M−H]^−^ via electrospray ionization mass spectrometry (ESI-MS) (Figure S1). The ^1^H nuclear magnetic resonance (NMR) spectrum of ACC-311 measured in deuterated methanol (CD_3_OD) exhibited signals due to a terminal methylene group at δ 4.70 (H-29) and 4.58 (H-29), two oxygenated methines at δ 3.60 (H-2) and 2.88 (H-3), six methyls at δ 1.69 (H-30), 1.00 (H-27), 0.98 (H-23), 0.96 (H-26), 0.91 (H-25), and 0.77 (H-24), and five methines and nine methylenes, which appeared at δ 0.70–3.70 (Table 2; Figure S2). Based on the ^13^C NMR and HMQC spectra results, 30 carbons were evident, including carboxyl carbon at δ 180.4 (C-28), sp^2^ quaternary carbon at δ 152.1 (C-20), a terminal methylene carbon at δ 110.1 (C-29), two oxygenated carbons at δ 84.4 (C-3) and 69.8 (C-2), as well as 25 carbons between δ 15.1 and 57.6, including six methyl carbons at δ 29.1 (C-23), 19.6 (C-30), 17.9 (C-25), 17.2 (C-24), 16.7 (C-26), and 15.1 (C-27), five quaternary carbons at δ 57.6 (C-17), 43.6 (C-14), 42.0 (C-8), 40.5 (C-4), and 39.5 (C-10), and five methine and nine methylene carbons (Table 2, Figure S3). The ^1^H and ^13^C NMR data of ACC-311 were identical to those of ALA. These spectral data were well-matched with previously published data [31,32]. The chemical structure was confirmed by the interpretation of two-dimensional NMR spectra, including ^1^H–^1^H correlation spectroscopy (^1^H–^1^H COSY) and heteronuclear multiple-bond correlation spectroscopy (HMBC, Figure 4 and Figure S4–S6). The purity of ACC-311 was 93.81% based on HPLC analysis (Figure S7).

### 2.3. Inhibitory Effects of Active Constituents on I_CRAC_ and Ca^2+^ Influx

**Figure 5:**
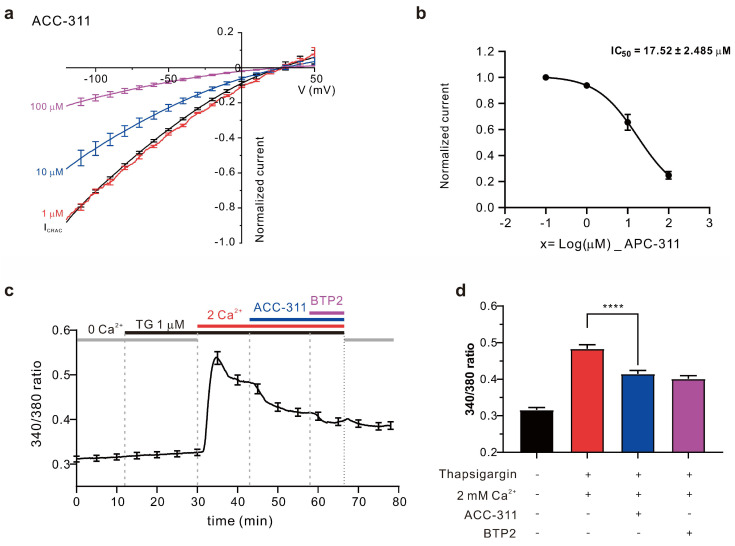
I_CRAC_ inhibition induced by ACC-311 causes inhibitory effects on Ca^2+^ influx in Jurkat T cells. (**a**) Current–voltage (IV) curve illustrating the inhibition of I_CRAC_ induced by ACC-311. (**b**) Half maximal inhibitory concentration (IC_50_) of ACC-311. Data are presented as mean ± SEM. (**c**) Intracellular calcium signaling measured with Fura-2 AM dye in Jurkat T cells. Calcium influx induced by 1 μM thapsigargin and reduced by 30 μM ACC-311. (**d**) Average ratio of calcium signaling reduced by 30 μM ACC-311. **** *p* < 0.0001.

Next, we analyzed the inhibitory effect of a single-type compound isolated from *A. coreana*, i.e., ACC-311, on I_CRAC_. The results revealed concentration-dependent I_CRAC_ inhibitory activities of ACC-311, leading to inhibition by 6.17 ± 1.057% at 1.0 μM, 34.46 ± 6.067% at 10.0 μM, and 75.22 ± 2.956% at 100 μM, whereas the calculated half maximal inhibitory concentration (IC_50_) was 17.52 ± 2.458 μM (Figure 5a,b). Considering that intracellular calcium influx through I_CRAC_ plays a major role in T cell activation and proliferation, we induced calcium influx through CRAC in Jurkat T cells to determine whether the I_CRAC_ inhibitory effect of ACC-311 reduces calcium influx. ER depletion was achieved via thapsigargin treatment while maintaining 0 mM calcium in the extracellular solution. Subsequently, 2 mM calcium was added to induce intracellular calcium influx. When the calcium influx was constant, 30 μM ACC-311 was added. Results confirmed suppression of calcium influx (Figure 5c,d).

### 2.4. Inhibitory Effects of Active Constituents on CD4^+^ T Cell Proliferation

**Figure 6:**
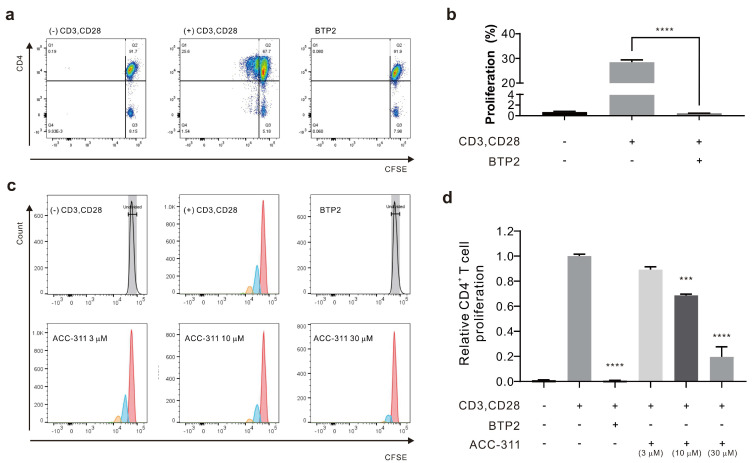
I_CRAC_ inhibition induced by ACC-311 causes inhibitory effects on human primary T cell proliferation. (**a**) Cell proliferation of human CD4^+^ T cells stained with CFSE and cultured for 3 days after stimulation with anti-CD3 and anti-CD28. Flow cytometry results of T cell proliferation before and after stimulation. BTP2, a CRAC inhibitor at 10 μM, served as the negative control. (**b**) Graph showing the proliferation rate of CD4+ T cells. (**c**) Inhibition of human primary CD4^+^ T cell proliferation by ACC-311. The control reagent used was an I_CRAC_ inhibitor, 10 μM BTP2. (**d**) Proliferation of primary CD4^+^ T cells based on ACC-311 concentration. *** *p* < 0.001, **** *p* < 0.0001.

To determine whether ACC-311 could suppress T cell proliferation via I_CRAC_ inhibition, CD4^+^ T cells were produced by stimulating human peripheral blood mononuclear cells (PBMCs) with anti-CD3 and anti-CD28 antibodies. To confirm T cell proliferation, the cells were stained with carboxyfluorescein diacetate succinimidyl ester (CFSE); cultured T cells were analyzed by flow cytometry after three days (Figure 5c). To verify the inhibitory effect on T cell proliferation, 3, 10, and 30 μM ACC-311 were applied and an ORAI1 inhibitor 3,5-bis(trifluoromethyl) pyrazole derivative (BTP2) served as the negative control. The level of CD4^+^ T cell proliferation was 0.69 ± 0.140% in the negative control group ((−)anti-CD3 and (−)anti-CD28), 28.47 ± 0.940% in the positive group ((+)anti-CD3 and (+)anti-CD28), and 0.45 ± 0.033% in the BTP2 group ((+)anti-CD3 and (+)anti-CD28 and BTP2) (Figure 6a,b). The ACC-311 fraction inhibited T cell proliferation in a dose-dependent manner by 10.78 ± 2.320% at 3 μM, 31.31 ± 1.011% at 10 μM, and 80.48 ± 8.109% at 30 μM (Figure 6c,d).

## 3. Discussion

The anti-inflammatory, antioxidant, and anti-viral effects of *A. coreana* have been demonstrated previously. Many studies have also investigated the diversity of constituents in this plant species [20]. Herein, a constituent of *A. coreana* exhibiting an anti-inflammatory effect was identified, and the anti-inflammatory effect was evaluated in terms of ion channel inhibition. According to a previous study, *A. pilosa* methanol extract and its fractions could inhibit ORAI1 [33]. In contrast, the current study obtained *A. coreana* extracts using 70% and 95% ethanol rather than methanol; the extraction yields and inhibitory effects were compared based on the EtOH content. Next, to identify the active component that exhibited an I_CRAC_ inhibitory effect, the extracts of *A. coreana* were fractionated with HEX, CHCl_3_, EtOAc, BuOH, and water. The CHCl_3_ fraction exhibited I_CRAC_ inhibition and was further separated via several columns. Ultimately, one constituent, i.e., ACC-311, was identified as ALA in the NMR analysis. To the best of our knowledge, among all studies reporting on the constituents of *A. coreana*, the present study is the first to report the isolation and identification of ALA.

We further assessed whether ACC-311 exerts an anti-inflammatory effect by regulating intracellular calcium signals through I_CRAC_ inhibition. The estimated IC50 for ACC-311 to inhibit I_CRAC_ was 17.52 ± 2.458 μM, and ACC-311 inhibited intracellular calcium influx through CRAC in Jurkat T cells. We thus posited that inhibition of intracellular calcium signaling would suppress T cell proliferation. To test this hypothesis, we evaluated the inhibitory effect of ACC-311 on human CD4+ T cell proliferation following stimulation with anti-CD3 and anti-CD28. Apparent inhibition was observed on cell proliferation. The I_CRAC_ inhibitor, BTP2, also effectively inhibited T cell proliferation. The inhibitory activity of ACC-311 on CRAC may be caused by STIM1 and ORAI1 regulation, two major proteins involved in CRAC activity. However, this study did not further elucidate whether the associated mechanism involves the ORAI1 pathway or STIM1, or interference in the formation of the ORAI1 and STIM1 complex. This will be a topic of a future study [34]. The role of CRAC in T cell activation and the CaMK/CaN/NFAT signaling pathway is well known [3,4]. Immunosuppressive drugs like cyclosporine A and tacrolimus, which have been clinically proven to be efficacious, target the same pathway by regulating the NFAT pathway through CaN inhibition [35,36]. CRACs play a crucial role in this NFAT pathway and have been suggested as a target for CaN/NFAT regulation. It can be assumed that ACC-311 inhibits T cell activity by suppressing NFAT activity, as intracellular calcium concentration decreases through CRAC inhibition. Indeed, altered intracellular calcium concentration can act as a crucial signal for T cell proliferation and activation [37,38,39,40]. In a previous study, ALA was shown to inhibit the nuclear factor-kappa B (NF-κB) pathway [41]. As with intracellular calcium signaling, the NF-κB pathway is associated with immune cell activity, proliferation, and differentiation, whereas the pathway proteins are engaged in activities such as epithelial cell differentiation or apoptosis [42]. Regulation of the NF-κB pathway in immune cells affects the production of various cytokines that control cellular proliferation and apoptotic differentiation. Hence, the inhibitory effect on T cells in the present study could have led to a simultaneous anti-inflammatory effect via inhibition of the NF-κB pathway.

The inhibitory effects of ALA on immune cell proliferation were mediated by inhibiting a CRAC. However, it remains uncertain whether ALA is the sole contributor to the anti-inflammatory effect of *A. coreana* as not all constituents of all fractions with a CRAC inhibitory effect were investigated. Hence, further investigation is needed to verify or expand our findings.

In summary, we isolated and purified a previously unreported ALA from *A. coreana* extract. Additionally, we demonstrated its inhibitory effect on I_CRAC_ and the associated suppression of intracellular calcium influx, resulting in inhibition of CD4^+^ T cell proliferation. CRAC inhibition is a mechanism that has not yet been targeted to achieve immunosuppression because it modulates upstream signaling pathways rather than inhibiting CaN or regulating the activity of NFAT. The ALA we discovered is a drug with these effects and can be effective in treating inflammation and allergic diseases caused by T cell activation.

## 4. Materials and Methods

### 4.1. Materials and Chemicals

All chemicals, unless otherwise specified, were purchased from Sigma-Aldrich Korea. Ficoll–Paque density gradient (GE Healthcare, Chicago, IL, USA), 1% penicillin/streptomycin (P/S; Hyclone, Logan, UT, USA), thapsigargin 1 μM (Sigma), pre-coated silica gel 60 F254 (0.25 mm; Merck, Darmstadt, Germany), silica gel 60 (0.063–0.200 mm; Merck), Sephadex LH-20 (bead size 25–100 µM; Pharmacia, Stockholm, Sweden), TSKgel ODS (i.d. 4.6 × 150 mm; Tosoh, Tokyo, Japan), and Dulbecco’s modified Eagle’s medium (DMEM; WelGene, Gyeongsan-si, Republic of Korea), fetal bovine serum (FBS; Welgene, Republic of Korea), Roswell Park Memorial Institute (RPMI)-1640 (Thermo Fisher Scientific, Waltham, MA, USA), Fura-2 acetoxymethyl ester (Fura-2 AM; Thermo Fisher Scientific), CD4+ T cell isolation kit (Miltenyi Biotec, Bergisch Gladbach, Germany), and 3,5-bis(trifluoromethyl) pyrazole derivative (BTP2; Merck, Germany) were used.

### 4.2. General Experimental Procedures

All solvents used for extraction and separation were of analytical grade (SK Chemicals) and HPLC solvents were of HPLC grade (Burdick & Jackson, Muskegon, MI, USA). Thin-layer chromatography (TLC) was carried out on pre-coated silica gel 60 F254 (0.25 mm) and reversed-phase TLC plates 60 F254 (0.25 mm). Column chromatography was performed over Sephadex LH-20 (bead size 25–100 µM; Pharmacia, Sweden) and silica gel 60 (0.063–0.200 mm;). MPLC was performed using a CombiFlash RF (Teledyne Isco, Lincoln, NE, USA). The HPLC system used for the isolation and analysis comprised a Hitachi L-2455 diode array detector, L-2130 HPLC pump, EZChrom Elite data system with a TSKgel ODS (i.d. 4.6 × 150 mm), and COSMOSIL C18 (i.d. 10 × 150 mm; Nacalai Tesque, Kyoto, Japan) columns. The NMR spectra were recorded at 500 MHz (^1^H) and 125 MHz (^13^C) on a JEOL JNM ECA-500 FT-NMR spectrometer (JEOL Ltd., Tokyo, Japan) in CD_3_OD. The ESI-MS spectra were measured using an Agilent 6410 Triple Quad LC/MS (Agilent Technologies, Santa Clara, CA, USA) spectrometer with a TSKgel ODS (Tosoh, Japan) (i.d. 4.6 × 150 mm).

### 4.3. Extraction and Isolation

The dried aerial parts of A. coreana were ground; 200 g of each sample was extracted with 20-fold (4 L) ethanol solvent (70%, 95%) for 4 h and filtered at room temperature using a 5 μm filter paper. The filtered samples were concentrated under reduced pressure at 45 °C and subsequently freeze-dried. The yield from 70% ethanol was 10.85%, resulting in 21.7 g of extract, while that of the 95% ethanol extract was 4.25% (8.5 g of extract).

The dried aerial part of A. coreana (254.3 g) was extracted with 70% aqueous EtOH at room temperature (25 °C). The extract was concentrated under reduced pressure, and the resultant aqueous fraction was sequentially partitioned using hexane, chloroform, and water. The CHCl3-soluble portion was concentrated under reduced pressure, subjected to silica gel column chromatography, and eluted stepwise with hexane:EtOAc (10:1–1:1, *v*/*v*). The active fraction A was further separated by Sephadex LH-20 column chromatography and eluted with CHCl3:MeOH (1:1, *v*/*v*) to produce an active fraction. This active fraction was chromatographed on a column of Sephadex LH-20 and eluted with methanol to create an active sub-fraction A1, which was subjected to reversed-phase MPLC and eluted with a gradient using increasing MeOH in water (30% to 60% aqueous MeOH) to produce the A1-1 subfraction. This sub-fraction was subjected to preparative HPLC using a system equipped with a COSMOSIL C18 (i.d. 10 × 150 mm) column with isocratic elution using 65% aqueous acetonitrile at a flow rate of 3 mL/min to yield ACC-311. To increase the purity of ACC-311, it was subjected to preparative HPLC using a system equipped with TSKgel ODS (i.d. 4.6 × 150 mm) column with a gradient elution using increasing acetonitrile in water (70% to 100% aq. acetonitrile) at a flow rate of 1 mL/min to yield ACC-311 (2.8 mg).

### 4.4. HPLC

The purity of ACC-311 (2.8 mg) was assessed using an HPLC system equipped with a TSKgel ODS (4.6 i.d. × 150 mm) column and photodiode array (Hitachi L-2455 diode array detector, Japan) and eluted with a gradient solvent system of 20% aq. acetonitrile to acetonitrile containing 0.04% trifluoroacetic acid at a flow rate of 1 mL/min.

### 4.5. Nuclear Magnetic Resonance Spectrometry

All NMR spectra were recorded on a JEOL JNM ECA500 FT-NMR spectrometer. The NMR measurements, including those from ^1^H, ^13^C, HMQC, HMBC, and ^1^H-^1^H COSY analyses, were carried out using 5 mm probe tubes at a temperature of 30 °C in CD_3_OD solutions. The ^1^H NMR and ^13^C NMR chemical shifts were referenced to the residual solvent peak of CD_3_OD at [δ_H_ 3.31, δ_C_ 49.0] ppm for ^1^H nucleus and ^13^C nucleus, respectively. All sample concentrations ranged from 2 to 4 mg/mL, with a total volume of 0.6 mL for each sample. The pulse conditions were as follows: ^1^H spectrum, spectrometer frequency (SF) = 500.16 MHz, acquisition time (AQ) = 2.6214 s, relaxation delay (RD) = 20 s, pulse width = 3.5, and spectral width (SW) = 12,500 Hz; ^13^C spectrum, SF = 125.77 MHz, AQ = 0.8284 s, RD = 2.0 s, pulse width = 3.6333, and SW = 39,557 Hz. Chemical shifts are reported in parts per million based on solvent signal.

### 4.6. Cell Culture

Human embryonic kidney 293 T cells (HEK293T cells; ATCC, Manassas, VA, USA) were cultured in DMEM containing 10% FBS and 1% P/S. The cells were sub-cultured once every two days in a 37 °C incubator at 10% CO_2_. HEK293T cells were seeded at a density of 7 × 10^5^ per 25T flask, and sub-cultured until they reached 80% confluence. The detailed protocol has been described previously [13]. Jurkat T cells were purchased from ATCC and cultured in RPMI-1640 with 10% FBS and 1% P/S. Jurkat T cells were sub-cultured by splitting 1/3 of the total cells every two days, and culturing in a 5% CO_2_ environment at 37 °C.

### 4.7. Electrophysiology

To obtain ORAI1 ion channel measurements, HEK293T cells with simultaneous transient transfection of ORAI1 and STIM1 were used. The details have been described previously [13].

### 4.8. Calcium Imaging

Ca^2+^ measurements were performed with Jurkat T cells in a solution with 145 mM NaCl, 3.6 mM KCl, 10 mM HEPES, 1 mM MgCl_2_, 1 mM EGTA, and 5 mM glucose, adjusted to 7.4 pH with NaOH. Jurkat T cells were incubated with Fura-2 AM (Thermo Fisher Scientific) at 2 μM final concentration for 30 min at 37 °C. These cells were loaded on 14 mm coverslips pre-coated with poly-L-Lysine. The ORAI1 channels were activated through store depletion induced by exposing thapsigargin 1 μM. Fura-2 AM was excited at wavelengths of 340 nm and 380 nm and collected at an emission wavelength of 510 nm. Fluorescence signals were measured with a digital system including an illuminator (pE-340 fura; CoolLED, Andover, UK) and a camera (sCMOS pco.edge 4.2; PCO, Kelheim, Germany). The 340/380 ratio was obtained every 10 s and analyzed using NIS-Element AR Version 5.00.00 (Nikon, Tokyo, Japan).

### 4.9. Human CD4^+^ T Cell Proliferation Assay

The PBMCs used to analyze the inhibition of T cell proliferation were isolated from human blood, which was separated into four layers using a Ficoll–Paque density gradient. Only the PBMC layer was collected. From the PBMCs, only CD4^+^ T cells were isolated and grown for assessment using the CD4^+^ T cell isolation kit. The details have been reported previously [16]. The present study was approved by the Institutional Review Board of Dongguk University College of Medicine (IRB No. 2017-07-003).

### 4.10. Statistics

In order to conduct statistical analysis, OriginPro 2021b (OriginLab Corporation, Northampton, MA, USA) and GraphPad Prism 8 (GraphPad Software Inc., San Diego, CA, USA) were employed for this study. All data are presented as mean ± standard error of mean (SEM). Sample comparisons were performed using the Student’s *t*-test, and the corresponding *p* values are indicated as follows: * *p* < 0.05, ** *p* < 0.01, *** *p* < 0.001, **** *p* < 0.0001.

## Figures and Tables

**Table 1:** Inhibition rates of the fractions from *A. coreana*.

Code Name	Inhibition Rate (%)
Fractionalized	
HEX	76.03 ± 5.654%
CHCl_3_	92.84 ± 1.901%
EA	43.31 ± 3.439%
BuOH	26.67 ± 11.930%
H_2_O	22.49 ± 6.832%
Silica gel column	
AC-C-3	50.96 ± 7.190%
AC-C-4	29.77 ± 4.434%
AC-C-5	21.65 ± 11.199%
AC-C-6	53.98 ± 9.776%
AC-C-7	63.91 ± 9.654%
Sephadex LH-20	
AC-C-3-1	18.16 ± 0.360%
AC-C-3-2	20.19 ± 8.809%
AC-C-3-3	25.40 ± 1.414%
AC-C-3-4	19.06 ± 6.094%
AC-C-3-5	17.21 ± 0.292%
ODS-MPLC	
MPLC1	58.32 ± 4.681%
MPLC2	38.36 ± 4.840%
MPLC3	12.04 ± 2.072%
MPLC4	81.86 ± 8.631%
MPLC5	18.22 ± 2.106%

**Table 2:** ^1^H (500 MHz) and ^13^C (125 MHz) NMR spectroscopic data for alphitolic acid (**1**) (δ_H_, mult., *J* in Hz).

No.	Alphitolic Acid (1)	
	δ_C_	δ_H_
1	48.4	1.99 (m), 0.83 (m)
2	69.8	3.60 (ddd, 11.5, 9.5, 5.0)
3	84.4	2.88 (d, 9.5)
4	40.5	
5	56.8	0.79 (m)
6	19.5	1.53 (m), 1.42 (m)
7	35.5	1.44 (m), 1.38 (m)
8	42.0	
9	52.0	1.38 (m)
10	39.5	
11	22.2	1.45 (m), 1.29 (m)
12	26.8	1.73 (m), 1.07 (m)
13	39.6	2.33 (td, 11.5, 9.0)
14	43.6	
15	30.8	1.54 (m), 1.16 (m)
16	33.4	2.23 (d, 13.0), 1.41 (m)
17	50.5	
18	39.6	1.61 (m)
19	48.5	3.03 (m)
20	152.1	
21	31.7	1.94 (m), 1.38 (m)
22	38.2	1.89 (m), 1.42 (m)
23	29.1	0.98 (s)
24	17.2	0.77 (s)
25	17.9	0.91 (s)
26	16.7	0.96 (s)
27	15.1	1.00 (s)
28	180.4	
29	110.1	4.70 (d, 2.0), 4.58 (d, 2.0)
30	19.6	1.69 (s)

## Data Availability

The data that support this study are available from the corresponding author upon reasonable request.

## Supplementary Materials

The following supporting information can be downloaded at: https://www.mdpi.com/article/10.3390/ijms242417309/s1.
