# Supplementary material for: Anti-Inflammatory Effects of Alphitolic Acid Isolated from Agrimonia coreana Nakai Extracts Are Mediated via the Inhibition of ICRAC Activity in T Cells
Source: Int J Mol Sci. 2023 Dec 9;24(24):17309. doi: 10.3390/ijms242417309 (PMC10743429; doi:10.3390/ijms242417309)
Supplement: Supplementary file 1 [file ijms-24-17309-s001.zip › ijms-2675089-supplementary.pdf]

Supporting Information

List of contents

Figure S1. ESI-MS spectrum (negative mode) of ACC-311 (**1**).

Figure S2. ^{1}H NMR spectrum of ACC-311 (**1**) in CD_3OD.

Figure S3. ^{13}C NMR spectrum of ACC-311 (**1**) in CD_3OD.

Figure S4. ^{1}H-^{1}H COSY spectrum of ACC-311 (**1**) in CD_3OD.

Figure S5. HMQC spectrum of ACC-311 (**1**) in CD_3OD.

Figure S6. HMBC spectrum of ACC-311 (**1**) in CD_3OD.

Figure S7. HPLC analysis of ACC-311 (**1**).

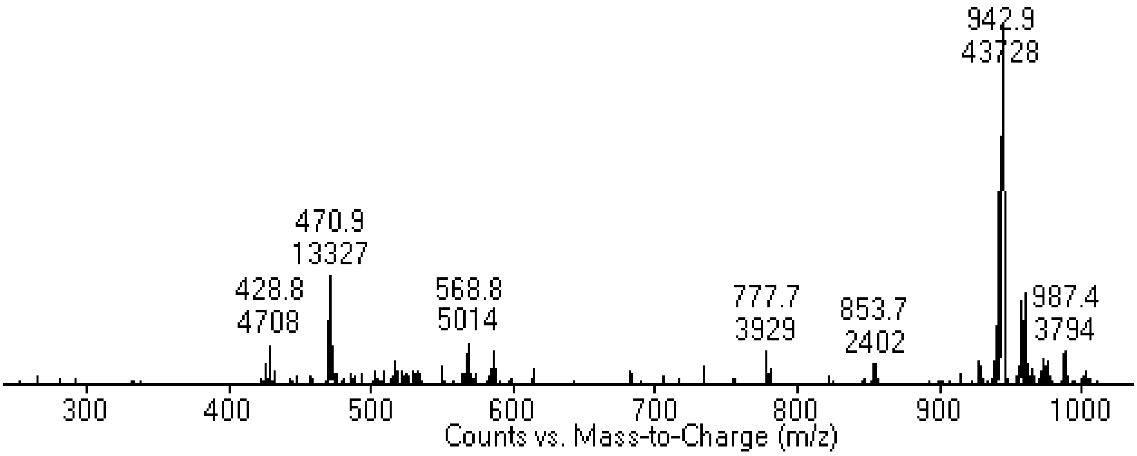

Figure S1. ESI-MS spectrum (negative mode) of ACC-311 (**1**).

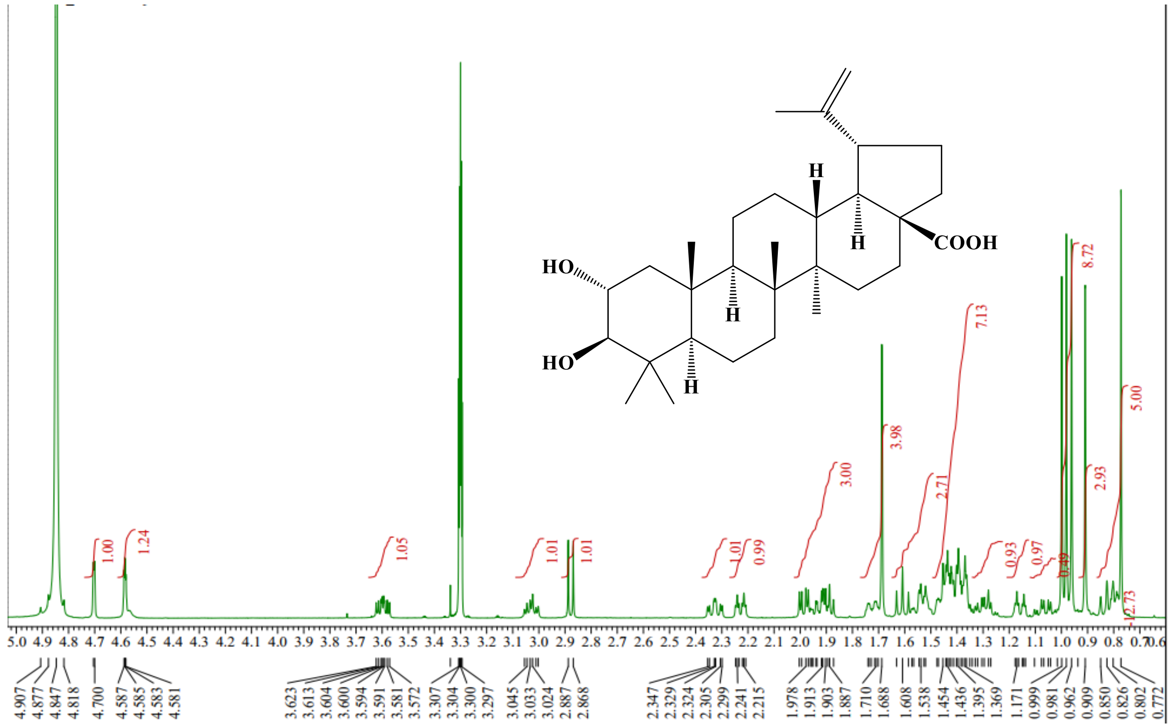

Figure S2. ^{1}H NMR spectrum of ACC-311 (**1**) in CD_3OD.

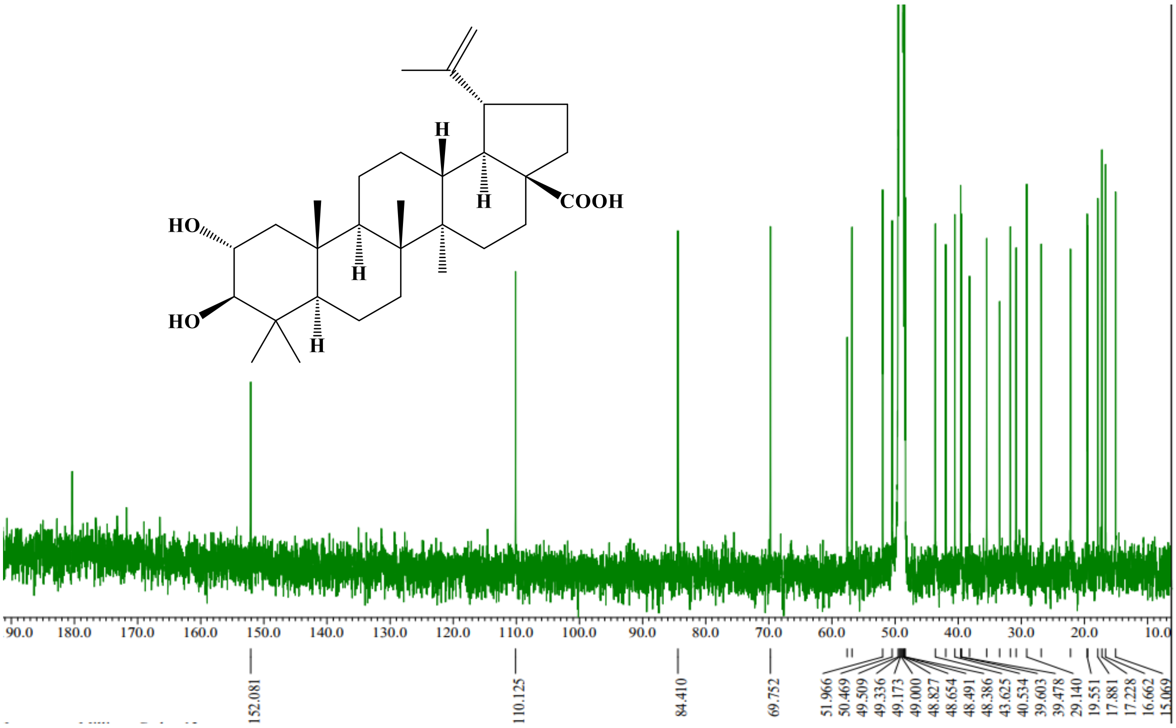

Figure S3. ^{13}C NMR spectrum of ACC-311 (**1**) in CD_3OD.

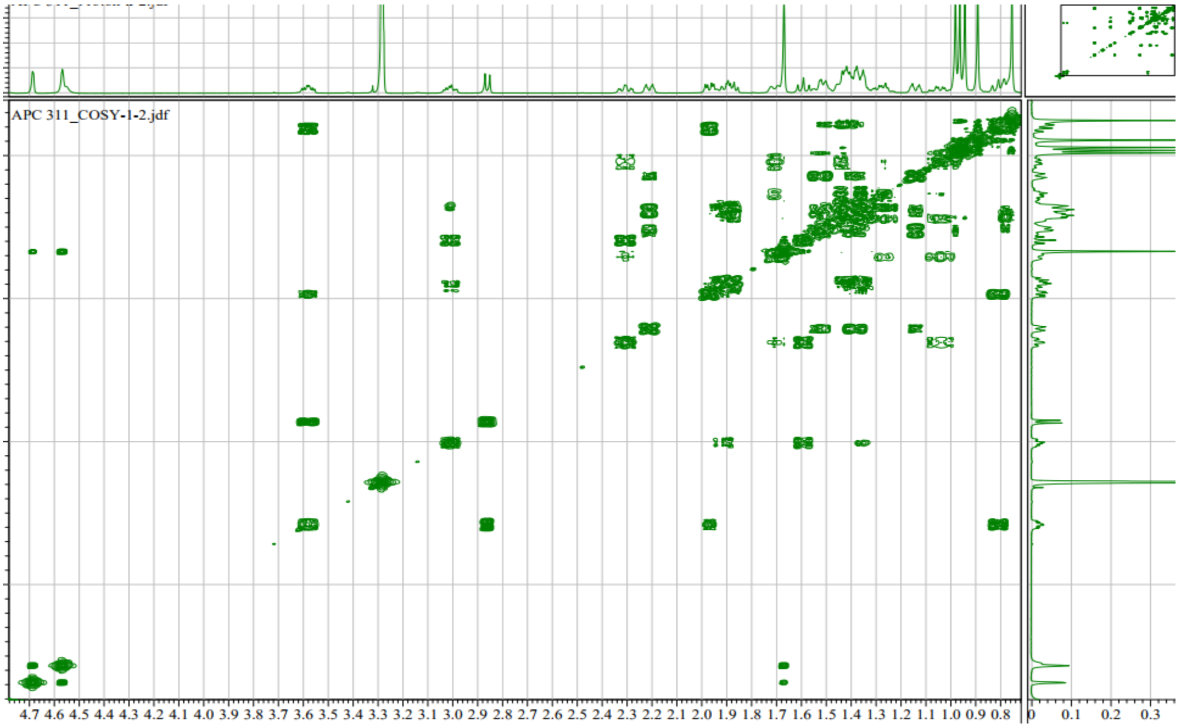

Figure S4. ^{1}H-^{1}H COSY spectrum of ACC-311 (**1**) in CD_3OD.

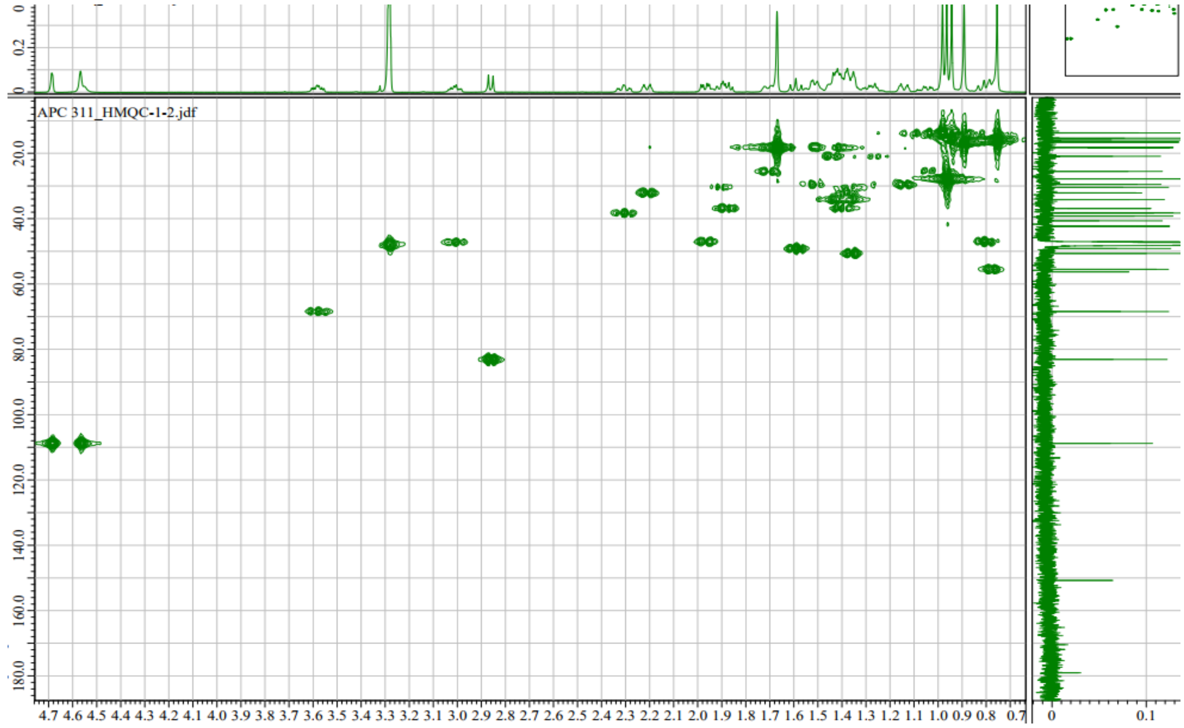

Figure S5. HMQC spectrum of ACC-311 (**1**) in CD_3OD.

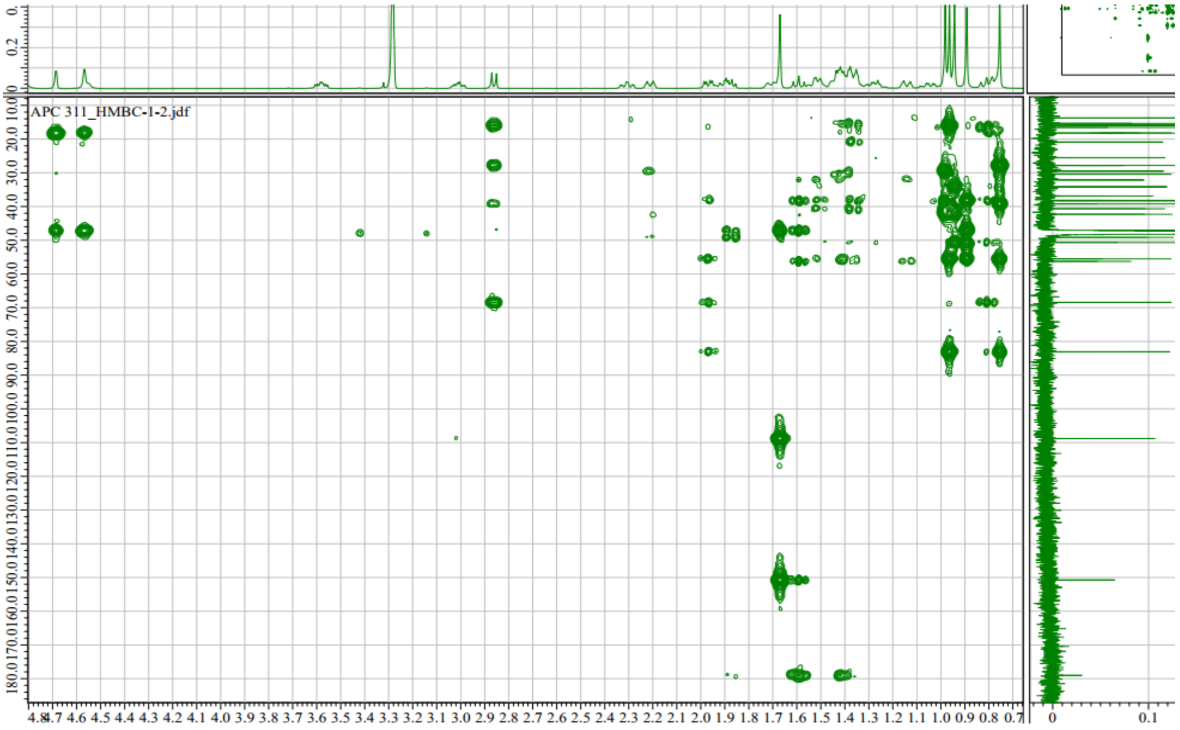

Figure S6. HMBC spectrum of ACC-311 (**1**) in CD_3OD.

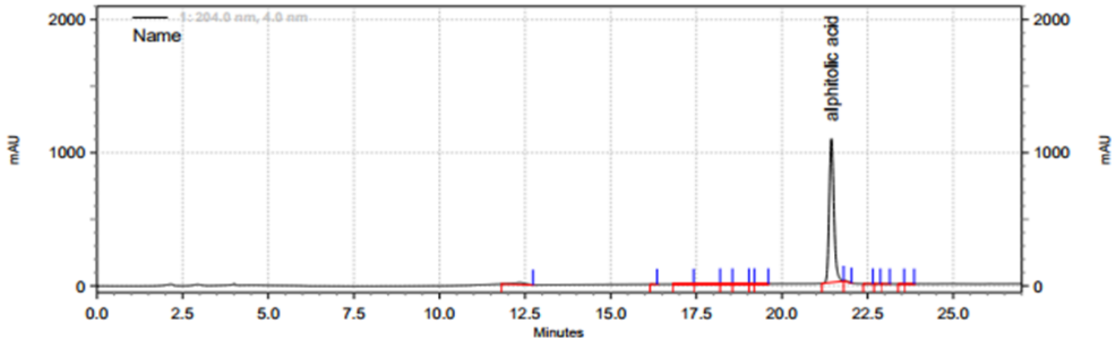

1: 204.0 nm, 4.0 nm
Results

Retention Time	Name	Area	Area %
21.447	**alphitolic acid**	41410039	93.81

Figure S7. HPLC analysis of ACC-311 (**1**)
